# Supplementary material for: S-SELeCT: a human-evolved serine integrase system for efficient large-cargo genome integration
Source: Nucleic Acids Res. 2026 Apr 2;54(6):gkag286. doi: 10.1093/nar/gkag286 (PMC13044945; doi:10.1093/nar/gkag286)
Supplement: gkag286_Supplemental_Files [file gkag286_supplemental_files.zip › Supplementary_Tables.pdf]

Supplementary Tables

Table S1. Variant 7 mutation summary.

Number	Mutation	AAs changed, deleted or inserted
1	EL -> FC at 162	2
2	Y -> KK at 173	2
3	L -> WV at 177	2
4	179 del SET	3
5	230 del PFK	3
6	233 ins LR	2
7	274 del PA	2
8	VM -> GTF at 277	3
9	EES -> PMW at 378	3
10	R389K	1

3.8% (23/605) AAs changed, deleted or inserted.

Table S2. Variant 12 mutation summary.

Number	Mutation	AAs changed, deleted or inserted
1	V114A	1
2	I116A	2
3	GVFRQGNVMDLI -> RALRRGSIADPV at 122	9
4	I136A	1
5	L139P	1
6	S142N	1
7	KES -> RGP at 144	3
8	RE -> AF at 161	2
9	A171R	1
10	Y -> KK at 173	2
11	L -> WV at 177	2
12	179 del SET	3
13	INKLAH -> A at 195	5
14	TT -> EPARSAA at 202	7
15	T206A	1
16	H228R	1
17	230 del PF	2
18	M251V	1

Number	Mutation	AAs changed, deleted or inserted
19	D254G	1
20	TR -> AW at 258	2
21	PA -> TG at 274	2
22	F289S	1
23	VIYKKK -> ATYRRG at 293	5
24	D300G	1
25	304 ins AG	2
26	306 del KIE	3
27	Y -> GC at 310	2
28	I312M	1
29	D315G	1
30	I317V	1
31	IE -> AG at 330	2
32	WY -> RH at 335	2
33	Q339R	1
34	D343G	1
35	G346D	1
36	K349A	1
37	355 ins V	1
38	IL -> P at 357	2
39	M361A	1
40	E367G	1
41	SK -> PE at 374	2
42	EESIKDSYRCRR -> PIRVEGGCGCGE at 378	11
43	K391G	1

15.5% (94/605) AAs changed, deleted or inserted.

Table S3. Variant 29 mutation summary.

Number	Mutation	AAs changed, deleted or inserted
1	E162F	1
2	A171R	1
3	Y -> KK at 173	2
4	L177W	1
5	F -> LY at 231	2
6	274 del PA	2
7	VM -> GTIF at 277	4

Number	Mutation	AAs changed, deleted or inserted
8	EES -> PMW at 378	3
9	R389K	1

2.8% (17/605) AAs changed, deleted or inserted.

Table S4. Variant 30 mutation summary.

Number	Mutation	AAs changed, deleted or inserted
1	161 del RELG	4
2	165 ins F	1
3	A171R	1
4	173 del YGF	3
5	L -> EW at 177	2
6	178 ins A	1
7	230 del PFK	3
8	233 ins LR	2
9	274 del PA	2
10	VM -> GTF at 277	3
11	EES -> PMW at 378	3
12	R389K	1

4.3% (26/605) AAs changed, deleted or inserted.

Table S5. Variant 32 mutation summary.

Number	Mutation	AAs changed, deleted or inserted
1	V114A	1
2	VS -> AG at 117	2
3	N128D	1
4	D131G	1
5	IH -> VR at 133	2
6	M137V	1
7	LD -> PG at 139	2
8	HK -> RR at 143	2
9	S146P	1
10	REL -> GFC at 161	3
11	173 del YGF	3
12	L -> EW at 177	2
13	178 ins A	1

Number	Mutation	AAs changed, deleted or inserted
14	INK -> LGG at 195	3
15	H200Y	1
16	T203A	1
17	LT -> PA at 205	2
18	H228R	1
19	PFKP -> R at 230	4
20	274 del PA	2
21	VM -> GTIF at 277	4
22	F289L	1
23	EVIYKKK -> RATHGEE at 292	7
24	D300G	1
25	TTKIE -> AAEVG at 304	5
26	I312A	1
27	D315G	1
28	I317T	1
29	321 del PVE	3
30	DC -> AGPGH at 325	5
31	IE -> AR at 330	2
32	W335R	1
33	EL -> GP at 337	2
34	L342S	1
35	345 ins GG	2
36	348 del GK	2
37	S352P	1
38	MDK -> VGG at 361	3
39	CE -> RG at 366	2
40	370 ins IT	2
41	MTS -> P at 372	3
42	EESIKD -> PMWVEG at 378	6
43	386 del RCR	3
44	389 ins Y	1
45	391 ins RE	2

16.2% (98/605) AAs changed, deleted or inserted.

Table S6. Variant 36 mutation summary.

Number	Mutation	AAs changed, deleted or inserted
1	REL -> GFD at 161	3
2	A171R	1
3	Y -> KK at 173	2
4	L -> WV at 177	2
5	179 del SET	3
6	H228R	1
7	230 del PF	2
8	274 del PA	2
9	276 ins GTI	3
10	EES -> PMW at 378	3
11	R389K	1

3.8% (23/605) AAs changed, deleted or inserted.

Table S7. Variant 37 mutation summary.

Number	Mutation	AAs changed, deleted or inserted
1	REL -> GFD at 161	3
2	A171R	1
3	Y -> KK at 173	2
4	L -> EWVA at 177	4
5	179 del SET	3
6	H228R	1
7	PF -> Y at 230	2
8	274 del PA	2
9	VM -> GTIF at 277	4
10	EES -> PMW 378	3
11	R389K	1

4.3% (26/605) AAs changed, deleted or inserted.

Table S8. Variant 40 mutation summary.

Number	Mutation	AAs changed, deleted or inserted
1	V114A	1
2	REL -> GFD at 161	3
3	Y -> KK at 173	2
4	177 del LVS	3
5	180 ins WVEV	4

Number	Mutation	AAs changed, deleted or inserted
6	H228R	1
7	F -> LY at 231	2
8	273 del DP	2
9	VM -> GTIF at 277	4
10	D300G	1
11	D315N	1
12	I317T	1
13	I330V	1
14	L358P	1
15	EES -> PMW at 378	3
16	R389K	1

5.1% (31/605) AAs changed, deleted or inserted.

Table S9. Summary of total AAs changed, deleted or inserted.

Variant	Total change count	Total change percent
V7	23	3.8%
V12	94	16.5%
V29	17	2.8%
V30	26	4.3%
V32	98	16.2%
V36	23	3.8%
V37	26	4.3%
V40	31	5.1%

Table S10. Sequences targeted using Cas9 for chromatin accessibility DNA-segment knock-in assay

**Locus code**	**Chromosome and band**	**Sequence of att-site candidate**	**SpCas9 target**	**PAM distance from att-site core [bp]**
A	4p14	GAGCATCCCCAACGAAGAGGACTTTCAGGTCTCCTTATTTGGGAAAACCC	CAAGAGCATCCCCAACGAAG	8
C	9q34.3	CTTATGCTCCAGGTGGATGCATGCACGAGTGTACCCAGCTGGGAGCTACC	CATGCACGAGTGTACCCAGC	14
D	11p12b	GCTGTGCCTGAAGTATGGGGAGATTTAAGTCCTCATAGTTAACTTGGAAC	GAAGTTCCAAGTTAACTATG	8
E	11q22.3	TTATATGTCTAACCCAGAAAAGTGACTGTTTTTCTCATTTAAGGAAATAA	GTAAAATGGCCTGGTTCTAA	147
